# Predisposing, enabling, and need factors associated with utilization of HCV testing services among PWID in two settings in India

**DOI:** 10.1186/s41124-016-0010-z

**Published:** 2016-06-29

**Authors:** Ruchi Sogarwal, Varada Madge, Pratyush Bishi, Apam Woleng, Rishi Garg

**Affiliations:** MAMTA Health Institute for Mother and Child, New Delhi, India

**Keywords:** Hepatitis C virus, People who inject drugs, India

## Abstract

**Background:**

The Hepatitis C virus (HCV) is very common among people who inject drugs (PWID), yet PWID in India have suboptimal access to HCV testing and treatment. This study sought to identify HCV risk factors among male PWID who utilized a free needle and syringe exchange program and to examine the predisposing, enabling, and need factors associated with utilization of HCV testing services by those PWID reporting that they had been tested.

**Methods:**

A cross-sectional study was conducted in Imphal, Manipur and Amritsar, Punjab. These two settings have high HCV prevalence and large numbers of PWID. A team of 18 field investigators obtained data through face-to-face interviews using a structured multiple-choice questionnaire. The questionnaire was administered to 1241 of 2644 male PWID aged 15 years and above enrolled in the needle and syringe program of India’s AIDS Control Program, with study participants selected through consecutive sampling. Statistical analyses included descriptive statistics and multivariate regression.

**Results:**

Twenty-four percent of PWID in our study sample reported having been tested for HCV. Unlike PWID in Imphal, more than half of PWID in Amritsar reported unprotected sex and use of alcohol or non-injecting drugs as being among their HCV risk factors (67.1 and 77.8 %, respectively). However, opioid substitution therapy non-adherence was reported more often in Imphal than in Amritsar. Education, marital status, place of residence and duration of injecting drug use were found to significantly enable access to HCV testing while alcohol use, frequent mobility and unprotected sex were found to significantly inhibit access to HCV testing for PWID after controlling for other explanatory variables.

**Conclusions:**

Predisposing and enabling determinants provide an area for developing effective interventions to improve HCV testing practices among PWID. HCV prevention programs that address safe injecting and sexual practices, OST adherence and frequent mobility customized for PWID by age are strongly recommended.

## Background

Hepatitis C virus (HCV) is a global public health problem, with around 150 million people chronically infected [1]. Approximately 2.7 % of all deaths annually are thought to be attributable to HCV [2]. This translates into one million deaths, most resulting from HCV-associated cirrhosis or HCV-associated liver cancer [3–5]. People who inject drugs (PWID) have disproportionately high HCV prevalence worldwide (50–90 %) in comparison to the general population [6]. Indian studies have reported HCV sero-positivity in PWID to be between 20 and 90 %. Some Indian PWID populations have extremely high HCV seroprevalence, while others have HCV seroprevalence in the more moderate range of 30 to 50 % [7–10]. Despite the high burden of disease in Indian PWID, the problem has not been addressed in a systematic way. Recently, national initiatives to increase case finding have been proposed, including recommendations for improved laboratory-based surveillance [11]. These initiatives are timely since recent treatment advances have resulted in most cases of chronic HCV being curable with the use of direct-acting antiviral regimens [12–14]. The prospects of translating these advances into population-level declines in HCV disease are currently limited by the fact that 50 to 75 % of HCV-infected individuals are unaware of their sero-status. Those under age 30 are particularly likely to be undiagnosed or to have experienced a late diagnosis [15–17].

In light of overlapping risk factors for transmission of viral hepatitis and HIV infection, as well as limited public health resources in many settings, the World Health Organization along with other experts has recommended integrating HCV and HIV services [18, 19]. In 2014, with the support of the Bristol-Myers Squibb Foundation [20], MAMTA Health Institute for Mother and Child [21] launched an initiative to reduce transmission of HCV and to improve patient care by integrating viral hepatitis prevention into existing public health programs providing HIV-related services to PWID in two settings in India: Amritsar District, in the state of Punjab, and Imphal District, in the state of Manipur. Historically, injection drug use was concentrated in the northeastern part of India, including Manipur, but rapidly growing populations of injection drug users have been reported in Punjab [22, 23]. Estimated HCV prevalence among PWID is 64.9 % in Imphal and 48.7 % in Amritsar [7].

The MAMTA intervention sought to empower people to seek health services early in regard to HCV screening, diagnosis and treatment, as well as to build the capacity of frontline health workers. It used the existing HIV program as a pathway for the expansion of viral hepatitis services, including vaccination, screening, confirmatory testing, and referral to care and treatment. In the first phase of the intervention, testing PWID for HCV was regarded as a key strategy and also an opportunity to promote health services and risk reduction services. However, program planners were hampered by a lack of knowledge about the characteristics of the population being targeted.

This study addresses the need for information in two ways. First, it investigates HCV risk factors among PWID utilizing a free needle and syringe exchange program. Second, it investigates which predisposing, enabling, and need factors were associated with utilization of HCV testing services by PWID. The conceptual framework for the study is based on the Gelberg-Andersen Behavioral Model for Vulnerable Populations [24], which has been extensively applied to the study of HIV testing in high-risk individuals [25–28]. *Predisposing* factors are demographic and other personal characteristics that influence the likelihood of obtaining care. *Enabling* factors are personal, family and community resources that support or encourage efforts to access health services. *Need* factors arise from the real or perceived need for health services and provide motivation for individuals to seek those services. In the absence of any similar research on HCV testing, we utilised the HIV literature to develop the hypothesis that are tested in this study. The hypothesis is that the predisposing factors would be associated with lower odds of utilisation of HCV testing as compared to enabling and need factors.

By comparing risk factors and HCV testing behavior in two settings that vary in regard to the nature of their drug use epidemics and HCV epidemics, this study also presents an opportunity to investigate whether different areas of India may benefit from different HCV prevention and testing approaches.

## Methods

### Study setting and participants

We surveyed male PWID utilizing a free multi-site needle and syringe exchange program in the districts of Amritsar and Imphal. Needle and syringe exchange took place at the community organization facilities of the Indian government’s State AIDS Control Program [29]. Facilities were selected to participate in the study if they met three criteria: willingness of facility managers; available financial resources; and more than 200 PWID enrolled for services. These criteria resulted in one out of nine facilities in Amritsar serving as a study site, along with five out of 23 facilities in Imphal. Consecutive individuals who were at least 15 years old and reported a history of injecting drug use were invited to participate in the study when they attended the needle and syringe exchange facilities at these six sites from April 2015 through July 2015.

### Instrument and variables

The data were obtained through face-to-face interviews by a team of 18 field investigators using a structured multiple-choice questionnaire that addressed prominent risk factors for HCV infection. The questionnaire included five domains relating to socio-demographic characteristics of the respondents, injecting patterns and practices, sexual practices, and other risk behaviors related to HCV including information about spouses and parents. The questionnaire was developed in English then translated into Hindi and Manipuri. All study participants were interviewed in the primary local language (Hindi in Amritsar and Manipuri in Imphal).

The main variable of interest (dependent variable) was self-report of ever receiving HCV testing. For the analysis of this study, we categorized independent variables into predisposing, enabling, and need factors in accordance with the previously described Gelberg-Andersen Behavioral Model for Vulnerable Populations [24]. Predisposing factors included age, education level, marital status, and use of alcohol and/or non-injecting drugs in the previous 1 month. Variables categorized as enabling factors included income status, ‘below-poverty-line’ status, level of mobility, district of residence, tattoo/piercing, unprotected sex in last 1 month, and sex with female sex workers in the last 1 month. Study participants were classified as belonging to either below-poverty-line (BPL) households or non-BPL households as defined by the government of India on the basis of three categories of vulnerability: residential, occupational and social [30]. Study participants were considered to be highly mobile if they spent more than 10 days per month interacting with other PWID at locations outside of the community where they resided [31]. The variables included as need factors were HCV-positive status of spouse or either parent; ever had transfusion of blood or blood components; ever had medical/dental surgery or hemodialysis; ever had needle stick injury; and sexually transmitted infection symptoms or treatment reported in last 1 month. Since frequency of injecting drug use is associated with likelihood of HCV transmission [32, 33], we included variables measuring current injecting drug use, injecting in groups in last 1 month, sharing needles/syringes and other equipment in last 1 month, and duration of injecting drug use.

### Statistical analysis

Descriptive analyses were used to assess the characteristics of study participants and the occurrence of HCV risk factors among younger PWID (aged 15–29) and older PWID (aged 30–44). In order to understand differentials in level of HCV risk factors by age and district of residence, ratios were computed. A ratio value of less than 1 would mean that the levels of risk factors were relatively higher for clients in the reference category compared to other clients, and a ratio value of more than 1 would mean the opposite, while a value of 1 would indicate no difference in the risk factors by age (reference category: <30 years) or district of residence (reference category: Amritsar). Bivariate analysis examined associations between the dependent variable and predisposing, enabling, and need factors. All variables that were significantly associated with the dependent variable were included in multivariate regression modeling. All *p*-values less than 0.05 were considered significant. Results are presented as odds ratios (ORs), with the relationship between exposure (independent variable) and an outcome (i.e. HCV testing) compared to no exposure. We reported higher odds (OR > 1) when this relationship is positive, and lower odds (OR < 1) when the relationship is negative. All statistical analyses were performed using SPSS 21.0 (IBM Corp, Armonk NY, 2012).

## Results

Among 2644 needle and syringe exchange users invited to participate in the study, 1241 agreed (46.9 %). Two hundred and eighty eight of the 1241 study respondents were HIV positive. The overall mean age of study participants was 33.4 years. Imphal had a much larger proportion of study participants aged 30 and above (81.2 %) than did Amritsar (37.3 %) (Table 1). Less than 10 % of study participants had no formal education, and most reported completing either primary education (22.0 %) or secondary education (55.9 %). Just over half of study participants reported currently being married, while 42.3 % had never been married. More than four-fifths of people were employed, including those who reported part-time employment and self-employment. More than three-quarters had a monthly income of 6000 Indian Rupees (INR) or less (38.3 %, <3000 INR; 41.9 %, 3001–6000 INR), and approximately one-third met the criteria for below-poverty-line status. Study participants in Amritsar had engaged in injecting drug use for a median of 3.0 years, while those in Imphal had engaged in injecting drug use for a median of 8.6 years.

**Table 1:** Sociodemographic characteristics of PWID in Amritsar and Imphal districts, India

Characteristics	Amritsar	Imphal	Total
	(*N* = 507)	(*N* = 734)	(*N* = 1241)
	*N* (%)	*N* (%)	*N* (%)
Age (years)			
15–19	15 (3.0)	11 (1.5)	26 (2.1)
20–29	303 (59.8)	127 (17.3)	430 (34.6)
≥ 30	189 (37.3)	596 (81.2)	785 (63.3)
Education			
No formal education	65 (12.8)	50 (6.8)	115 (9.3)
Primary (up to 5 years of schooling)	174 (34.3)	99 (13.5)	273 (22.0)
Secondary (up to 10 years of schooling)	249 (49.1)	445 (60.6)	694 (55.9)
Higher (up to 12 years of schooling or beyond)	19 (3.7)	140(19.1)	159 (12.8)
Marital status			
Currently married	232 (45.8)	404 (55.0)	636 (51.2)
Widowed, divorced or separated	19 (3.7)	61(8.3)	80 (6.4)
Never married	256 (50.5)	269 (36.6)	525 (42.3)
Employment status			
Employed (including part-time employment and self-employment)	393 (77.5)	625 (85.1)	1018 (82.0)
Not employed	114 (22.5)	109 (14.9)	223 (18.0)
Monthly income (Indian Rupees)			
≤ 3000	122 (24.1)	353 (48.1)	475 (38.3)
3001–6000	270 (53.3)	250 (34.1)	520 (41.9)
6001–10,000	75 (14.8)	55 (7.5)	130 (10.5)
> 10,001	40 (7.9)	76 (10.4)	116 (9.3)
Below-poverty-level (BPL) status^a^			
Not below poverty level	383 (75.5)	438 (59.7)	821 (66.2)
Below poverty level	124 (24.5)	296 (40.3)	420 (33.8)
Age at sexual debut (years)			
Mean ± SD	18.0 ± 2.8	23.7 ± 5.7	21.2 ± 5.5
Median (range)	18.0 (10–31)	24.0 (10–48)	20.0 (10–48)
Duration of injecting drug use (years)			
≤ 1	130 (25.6)	42 (5.7)	172 (13.9)
2–5	292 (57.6)	137 (18.7)	429 (34.6)
6–10	49 (9.7)	190 (25.9)	239 (19.3)
≥ 11	36 (7.1)	365 (49.7)	401 (32.3)
Mean ± SD	±4.4	13.3 ± 11.0	9.5 ± 8.5
Median (range)	3.0 (1–35)	8.6 (1–35)	7 (1–35)

^a^BPL status was determined in accordance with the definition used by the Indian government (Planning Commission, Government of India: Report of the expert group to review the methodology for measurement of poverty; June 2014; http://planningcommission.nic.in/reports/genrep/pov_rep0707.pdf)

### Self-reported HCV risk factors

Findings for self-reported HCV risk factors were disaggregated by both place of residence (Amritsar versus Imphal) and age (<30 years versus ≥30 years) (Table 2).

**Table 2:** HCV risk factors reported by younger (<30 years) and older (≥30 years) study participants, by place of residence (*N* = 1241)

Reported HCV risk	Amritsar (*N* = 507)		Imphal (*N* = 734)		Ratio (≥30 years/ <30 years)		Ratio (IMP/AMR)	
	<30 years	≥30 years	<30 years	≥30 years	AMR	IMP	<30 years	≥30 years
	(*n* = 318 )	(*n* = 189)	(*n* = 138 )	(*n* = 596 )				
Daily injector in last 1 month	36/311 (11.6)	12/ 188 (6.4)	37/129 (28.7)	178/518 (34.4)	0.6	1.2	2.5	5.4
More than 30 injecting episodes per month in last consecutive 3 months	24/153 (15.7)	10/73 (13.7)	46/104 (44.2)	177/369 (47.9)	0.9	1.1	3.2	3.4
Injecting in a group in last 1 month	134/314 (42.7)	57/188 (30.3)	55/126 (43.7)	153/502 (30.5)	0.7	0.7	1.0	1.0
Sharing of needles/ syringes and other equipment in last 1 month	77/313 (24.6)	32/189 (16.9)	17/126 (13.4)	62/516 (12.0)	0.7	0.9	0.5	0.7
Unprotected sex in last 1 month	186/277 (67.1)	131/167 (78.4)	38/129 (29.5)	275/531 (51.8)	1.2	1.8	0.4	0.7
Sex with female sex worker in last 1 month	42/314 (13.4)	13/188 (6.9)	4/133 (3.0)	11/578 (1.9)	0.5	0.6	0.2	0.3
STI symptoms or STI treatment in last 1 month	10/315 (3.2)	3/189 (1.6)	1/132 (0.8)	4/571 (0.7)	0.5	0.8	0.2	0.4
Use of alcohol and/or other non-injecting drugs in last 1 month	245/315 (77.8)	177/189 (77.8)	77/133 (57.9)	284/579 (49.1)	1.0	0.8	0.7	0.6
Opioid substitution therapy non-adherence in last 1 month	71/315 (22.5)	30/189 (15.8)	71/132 (53.8)	348/580 (60.0)	0.7	1.1	2.4	3.8
High mobility in last 1 month^a^	26/307 (8.5)	10/187 (5.3)	28/132 (21.2)	110/577 (19.1)	0.6	0.9	2.5	3.6
Mother, father or spouse ever tested HCV-positive	1/314 (0.3)	0/189 (0)	3/131 (2.3)	18/572 (3.1)	0.0	1.3	7.7	-
Ever had tattoo or piercing	102/312 (32.7)	46/188 (24.5)	35/131 (26.7)	72/581 (12.4)	0.7	0.5	0.8	0.5
Ever had transfusion of blood or blood components	18/314 (5.7)	15/189 (7.9)	1/132 (0.8)	18/579 (3.1)	1.4	3.9	0.1	0.4
Ever had medical or dental surgery, or hemodialysis	13/315 (4.1)	25/189 (13.2)	1/131 (0.8)	16/582 (2.7)	3.2	3.4	0.2	0.2
Ever had needle stick injury	0/315	1/188 (0.5)	3/130 (2.3)	10/573 (1.7)	-	0.7	-	3.4

^a^High mobility for PWID is defined as spending more than 10 days per month interacting with other PWID at locations outside of the community where one resides

The Amritsar study cohort had more people under age 30 than age 30-plus who reported injecting daily in the last 1 month (11.6 versus 6.4 %; ratio: 0.6). The inverse relationship was seen in Imphal, where 28.7 % of people under age 30 and 34.4 % of people age 30-plus reported injecting daily in the last 1 month (ratio: 1.2). A similar pattern was seen for non-adherence to opioid substitution therapy. More Amritsar study participants under age 30 than age 30-plus reported non-adherence (22.5 versus 15.8 %; ratio: 0.7), while more Imphal study participants age 30-plus reported non-adherence (60.0 %) in comparison to the under-30 Imphal cohort (53.8 %) (ratio: 1.1)

In both Amritsar and Imphal, there was more reported sharing of injecting equipment in the last 1 month among under-30 study participants than among those age 30-plus, although the difference was greater in Amritsar (24.6 versus 16.9 %; ratio: 0.7). Similarly, larger proportions of under-30 study participants in both locations reported “ever had tattoo or piercing” as a risk factor. Regarding a third HCV risk factor, unprotected sex in the last 1 month, both locations had larger proportions of older study participants than younger study participants reporting that they had engaged in this behavior (Amritsar: <30 years, 67.1 %; ≥30 years, 78.4 %; ratio: 1.2; Imphal: <30 years, 29.5 %; ≥30 years, 51.8 %; ratio: 1.8).

When study participants under age 30 in Imphal were compared to study participants under age 30 in Amritsar, various differences were observed. A much larger proportion of the Imphal cohort than the Amritsar cohort reported injecting daily in the last month (28.7 versus 11.6 %; ratio: 2.5). On the other hand, smaller proportions of younger study participants in Imphal than in Amritsar reported other risk factors such as sharing of injecting equipment in the last 1 month (13.4 versus 24.6 %; ratio: 0.5), unprotected sex in the last month (29.5 versus 67.1 %; ratio: 0.4), and ever had tattoo or piercing (26.7 versus 32.7 %; ratio: 0.8). Regarding OST non-adherence in the last 1 month, 22.5 % of study participants under age 30 in Amritsar reported non-adherence, while 53.8 % of those under age 30 in Imphal did so (ratio: 2.4).

The age 30-plus study cohort had a similar pattern in risk factors across the two locations. A much larger proportion of older Imphal study participants reported being daily injectors in the last month in comparison to their counterparts in Amritsar (34.4 versus 6.4 %; ratio: 5.4). A much smaller proportion of older Amritsar study participants reported OST non-adherence in the last 1 month in comparison to the older Imphal cohort (15.8 versus 60.0 %; ratio: 3.8). Several other risk factors were reported by larger proportions of age 30-plus Amritsar residents than age 30-plus Imphal residents, including sharing of injecting equipment in the last 1 month (16.9 versus 12.0 %; ratio: 0.7); unprotected sex in the last 1 month (78.4 versus 51.8 %; ratio: 0.7); and ever had tattoo or piercing (24.5 versus 12.4 %; ratio: 0.5).

#### Factors associated with utilization of HCV testing

Two hundred and ninety-eight of the 1241 study participants (24.0 %) reported that they had been tested for HCV.

Three of four predisposing factors were significantly associated with HCV testing (Table 3). Study participants with any formal education were more likely to report testing than those with no formal education (adjusted odds ratio [aOR] 3.5, 95 % confidence interval [CI] 1.2–10.4, *p* = 0.022). Currently married study participants were more likely to report testing than those who were not (aOR 2.4, CI 1.3–4.5, *p* = 0.004). Study participants who reported using alcohol and/or other non-injecting drugs in the last 1 month were less likely than those who did not to report testing (aOR 0.6, CI 0.4–0.9, *p* = 0.016). As for the fourth predisposing factor, 9.7 % of study participants under age 30 and 32.2 % of study participants age 30-plus reported testing, but the older age group did not have significantly higher odds of testing (aOR 1.4, CI 0.8–2.3, *p* = 0.208).

**Table 3:** Univariate and multivariate logistic regression analyses of factors associated with previous HCV testing among PWID (*N* = 1241 unless otherwise noted)

Factors	Tested for HCV (%)	Univariate		Multivariate	
		OR (95 % CI)	*P*-value	Adjusted OR (95 % CI)	*P*-value
*Predisposing factor*					
Age (years)					
*< 30*	45/456 (9.7)	1	<0.001	1	0.208
*≥ 30*	253/785 (32.2)	4.4 (3.1–6.2)		1.4 (0.8–2.3)	
Education					
*No formal education*	13/115 (11.3)	1	0.001	1	0.022
*Formal education (any)*	285/1126 (25.4)	2.7 (1.5–4.8)		3.5 (1.2–10.4)	
Marital Status					
*Widowed, divorced, separated or never married*	112/605 (18.5)	1	<0.001	1	0.004
*Currently married*	186/636 (29.2)	1.8 (1.4–2.4)		2.4 (1.3–4.5)	
Use of alcohol and/or other non-injecting drugs in last 1 month (*N* = 1186 )					
*No*	162/433 (37.4)	1	<0.001	1	0.016
*Yes*	129/753 (17.1)	0.4 (0.3–0.5)		0.6 (0.4–0.9)	
*Enabling factor*					
District of residence					
*Amritsar*	28/507 (5.5)	1	<0.001	1	<0.001
*Imphal*	270/734 (36.8)	10.1 (6.7–15.2)		4.9 (2.7–8.8)	
Employment status					
*Not employed*	263/1018 (25.8)	1		1	0.802
*Employed*	35/223 (15.7)	0.5 (0.4–0.8)	0.002	1.1 (0.6–1.8)	
Below-poverty-level (BPL) status^a^					
*Not below poverty level*	157/821 (19.1)	1	<0.001	1	0.598
*Below poverty level*	141/420 (33.6)	2.1 (1.6–2.8)		1.1 (0.8–1.6)	
Unprotected sex in last 1 month (*N* = 1104)					
*Always used condoms for sex*	131/474 (27.6)	1	0.05	1	0.015
*Did not always use condoms for sex*	143/630 (22.7))	0.8 (0.6–1.0)		0.5 (0.3–0.9)	
High mobility in last 1 month^b^ (*N* = 1203)					
*No*	253/1029 (24.5)	1	0.099	1	0.005
*Yes*	33/174 (18.9)	0.7 (0.5–1.0)		0.5 (0.3–0.8)	
Sex with female sex worker in last 1 month (*N* = 1213)					
*No*	59/1143 (5.2)	1	0.106	1	0.195
*Yes*	11/70 (15.71)	0.6 (0.3–1.1)		2.1 (0.7–6.8)	
Ever had tattoo or piercing (*N* = 1212)					
*No*	238/957 (24.8)	1	0.083	1	0.259
*Yes*	50/255 (19.6)	0.7 (0.5–1.0)		1.3 (0.8–2.2)	
*Need factor*					
STI symptoms or STI treatment in last 1 month (*N* = 1207)					
*No*	280/1189 (23.5)	1	0.345	1	0.170
*Yes*	6/18 (33.3)	1.6 (0.6–4.3)		3.9 (0.5–27.2)	
Current injecting drug user (*N* = 1211)					
*No, but formerly*	131/512 (25.6)	1	0.163	1	0.525
*Yes, currently (injected drugs in last 3 months)*	155/699 (22.2)	0.7 (0.6–1.0)		0.9 (0.6–1.3)	
Injecting in a group in last 1 month (*N* = 1130)					
*No*	171/731 (23.4)	1	0.009	1	0.619
*Yes*	67/399 (16.8)	0.7 (0.5–1.1)		1.1 (0.7–1.8)	
Sharing of needles/syringes and other equipment in last 1 month (*N* = 1144)					
*No*	214/956 (22.4)	1		1	0.365
*Yes*	34/188 (18.1)	0.7 (0.5–1.1)	0.179	1.3 (0.7–2.1)	
Duration of injecting drug use (years)					
*≤ 1*	21/172 (12.2)	1		1	0.021
*2–5*	46/429 (10.7)	0.8 (0.5–1.5)	0.585	0.9 (0.5–1.9)	0.861
*6–10*	60/239 (25.1)	2.4 (1.4–4.1)	0.001	1.3 (0.6–2.7)	0.491
*≥ 11*	171/401 (42.6)	5.4 (3.3–8.9)	<0.001	2.1 (1.0–4.2)	0.050

^a^BPL status was determined in accordance with the definition used by the Indian government (Planning Commission, Government of India: Report of the expert group to review the methodology for measurement of poverty. June 2014; http://planningcommission.nic.in/reports/genrep/pov_rep0707.pdf)

^b^High mobility for PWID is defined as spending more than 10 days per month interacting with other PWID at locations outside of the community where one resides

Three of seven enabling factors were significantly associated with HCV testing. Residents of Imphal were much more likely than residents of Amritsar to report testing (aOR 4.9, CI 2.7–8.8, *p* < 0.001). Study participants who reported using condoms in the last 1 month were less likely to report testing than those who did not (aOR 0.5, CI 0.3–0.9, *p* = 0.015), as were those who reported high mobility in the last 1 month in comparison to those who did not (aOR 0.5, CI 0.3–0.8, *p* = 0.005).

Among five need factors, only one was significantly associated with HCV testing. Study participants who had been injecting drugs for 11 or more years were more likely to report testing than those who had been injecting for 1 year or less (aOR 2.1, CI 1.0–4.2, *p* = 0.050).

## Discussion

This cross-sectional study investigated prevalence of HCV risk factors among 1241 male PWID utilizing a free needle and syringe exchange program in two distinct settings in India, as well as assessing which factors were associated with utilization of HCV testing services. Study participants in Imphal, which has had an injection drug epidemic for longer than Amritsar, reported a much longer median number of years of injection drug use than their Amritsar counterparts. When reported HCV risk factors were compared across the study sites with study participants disaggregated into two age groups, some risk factors appeared to be more prominent among Amritsar PWID and other risk factors appeared to be more prominent among Imphal PWID regardless of age. For example, higher proportions of Amristar PWID in both age groups reported sharing needles/syringes and other injecting equipment in the last month, while higher proportions of Imphal PWID in both age groups experiencing OST non-adherence in the last month. Some risk factors also followed a consistent pattern across the two cities in relation to age. For example, higher proportions of PWID under age 30 in both cities reported injecting in groups in the last 1 month. At the same time, the two age groups exhibited different patterns of risk in different locations in certain regards. For example, more Amritsar PWID under age 30 had non-adherence to OST in the last month in comparison to older PWID in the same location, while older Imphal PWID reported this risk factor more often than did younger Imphal PWID. Among the subgroup of 298 study participants who reported previous HCV testing, Imphal residents were far more likely to do so than Amritsar residents, while age on the other hand was determined to not be a significant predictor of previous testing.

This study, by separately examining the potential influence of age and place of residence on PWID in relation to HCV risk, has provided evidence that some HCV-related needs vary considerably across different Indian PWID subpopulations. One factor that may help to account for this variation is the longevity of the injecting drug use epidemic. Two HCV risk factors reported by higher proportions of Amritsar residents than Imphal residents were sharing injection equipment and having unprotected sex. We speculate that Amritsar with its newer injection drug epidemic may lag behind Imphal in regard to the provision of a range of services for PWID, including interventions to reduce the sharing of injection equipment and promote condom use. At the same time, PWID in a setting with a longtime drug use epidemic, such as Imphal, may also have specific unmet needs. In our study, both younger and older PWID in Imphal, unlike their counterparts in Amritsar, reported quite high levels of non-adherence to opioid substitution therapy. Since the provision of OST is regarded as a key component of a comprehensive approach to reducing HCV transmission and preventing HCV reinfection among PWID, our study finding points to a problem that should be addressed through geographically targeted interventions. Furthermore, the finding that OST non-adherence was somewhat higher among older Imphal PWID (60.0 %) compared to younger Imphal PWID (53.8 %) suggests that age-targeted interventions addressing this issue might be beneficial in Imphal as well. One of the examples of the age-targeted intervention is the UFO (U Find Out) model for HCV prevention, a youth centered, collaborative and harm reduction based intervention, in which young IDU centered referral services are provided for early screening of HCV [34].

One-fourth of the PWID in our study reported ever being tested for HCV. In multivariate analysis, Imphal residents were almost five times as likely as Amritsar residents to report previous HCV testing. Nonetheless, when HCV testing findings were disaggregated by place of residence, the reported HCV testing level among Imphal residents was still only 36.8 %. Numerous factors may account for this disparity, such as diversity in drug use, epidemic stage of HCV and socio-demographic and risk behaviours [7, 8]. Additionally, prevention services such as the needle and syringe exchange are more numerous and accessible to Imphal residents; and PWID in this area may therefore have greater knowledge of available resources. There may be unmet needs for outreach services and a paucity of trained health care providers for PWID in less epidemic area. These results illustrate the need for expanded access to primary health care and prevention services that could be an important strategy to address an unmet need for individuals at high risk for HCV or detecting previously undiagnosed cases of HCV. For PWID who are not routinely engaged in medical care, NSEP may also be utilized resource for HCV screening.

Guided by Gelberg-Andersen model [24], we categorized multi-level factors that may facilitate or hinder HCV testing behavior of PWIDs. Contrary to our hypothesis, we found that most predisposing factors were associated with higher odds of utilisation of HCV testing as compared to enabling and need factors. This indicates that access to comprehensive services by PWID relates to socio-demographic factors. In the final adjustment model, education, marital status, place of residence and duration of injecting drug use were found to significant enable uptake of HCV testing services while alcohol use, mobility and unprotected sex were found to significantly inhibit uptake to HCV testing services. This indicates that targeting and reaching PWID who may be in need of HCV testing is complex, and cannot be determined by one element of vulnerability. This finding, coupled with the finding about variation in HCV risk factors across different age groups and settings, raises concern about an important dimension of the response to HCV in large heterogeneous countries such as India. National policies in India are translated into strategies and action plans at the state level. In order to develop an effective overall response, it is important to know which factors should be the highest-priority targets for interventions at the state and local levels. Multiple domains of information, including epidemiology as well as knowledge of program, geographic, and community settings, may be essential to develop effective interventions.

This study has several limitations. First, this sample was drawn from a group of organizations involved in implementing a specific National AIDS Control Program activity, and results cannot be assumed to represent PWID who are not receiving the same services. Second, study participants were recruited from the client rosters of the needle and syringe exchange facilities, and the dearth of female clients is reflected in the all-male composition of the study cohort. Study results thus do not reflect the experience of an important but often hidden segment of India’s injection drug-using population. Results are further limited by fact that study participants were from two districts in different Indian states. Whether findings can be generalized to PWID in other parts of those states or other parts of the country remains to be determined. Additionally, we could not verify the accuracy of self-reported data on HCV testing. Some study participants may have answered this question incorrectly due to recall bias, social desirability bias or some other reason. The data that were collected provided little insight into system-level factors that may affect HCV testing uptake, such as funding, regulation and service delivery infrastructure. Despite these limitations, the study findings may inform comprehensive interventions for systems delivering care to specific high-risk groups. Our findings are important because they suggest identifiable characteristics that can be targeted to reduce the risk and spread of HCV infection.

## Conclusion

Our findings highlight important factors that may be useful to increase HCV testing rates among PWID, which may strengthen prevention and reduce transmission of the infection. This study concludes that predisposing and enabling determinants provide an area for developing effective interventions to improve HCV testing practices and risk reduction. Prevention programs that address safe injecting and sexual practices, adherence to OST and frequent mobility customized for PWID by ‘age’ is strongly recommended to prioritize HCV risk reduction strategies. The priority factors found in this study coincide with the HIV prevention program in the country; hence integration of HCV within the harm reduction program could be a possible solution to address the HCV burden among PWID.

## Abbreviations

BPL, Below Poverty Line; HCV, Hepatitis C Virus; HIV, Human Immunodeficiency Virus; NGO, Non-government Organization; NSEP, Needle Syringe Exchange Program; OST, Opioid Substitution Therapy; PWID, People who Inject Drugs; STI, Sexually Transmitted Infection; TI, Targeted Intervention
